# A community-driven and evidence-based approach to developing mental wellness strategies in First Nations: a program protocol

**DOI:** 10.1186/s40900-020-0176-9

**Published:** 2020-02-12

**Authors:** Melody Morton Ninomiya, Ningwakwe (Priscilla) George, Julie George, Renee Linklater, Julie Bull, Sara Plain, Kathryn Graham, Sharon Bernards, Laura Peach, Vicky Stergiopoulos, Paul Kurdyak, Gerald McKinley, Peter Donnelly, Samantha Wells

**Affiliations:** 1 Centre for Addiction and Mental Health, Toronto and London, Ontario Canada; 2 Health Sciences, Wilfrid Laurier University, Waterloo, Ontario Canada; 3 Kettle & Stony Point Health Centre, Kettle & Stony Point First Nation, Toronto, Ontario Canada; 4 E’Mino Bmaad-Zijig Gamig, Aamjiwnaang First Nation Health Centre, Aamjiwnaang First Nation, Toronto, Ontario Canada; 5 Dalla Lana School of Public Health, University of Toronto, Toronto, Ontario Canada; 6 School of Psychology, Faculty of Health, Deakin University, Melbourne, Victoria Australia; 7 National Drug Research Institute, Curtin University, Perth, Western Australia Australia; 8 Centre for Urban Health Solutions, Li Ka Shing Knowledge Institute, St. Michael’s Hospital, Toronto, Ontario Canada; 9 Department of Psychiatry, University of Toronto, Toronto, Ontario Canada; 10 Institute for Clinical Evaluative Science, Toronto, Ontario Canada; 11 University of Western Ontario, London, Ontario Canada

**Keywords:** First Nation, Indigenous, Mental health, Substance use, Violence, Mental wellness, Community wellness, Community-based research, Participatory action research, Resilience

## Abstract

**Background:**

Mental health, substance use/addiction and violence (MSV) are important issues affecting the well-being of Indigenous People in Canada. This paper outlines the protocol for a research-to-action program called the Mental Wellness Program (MWP). The MWP aims to increase community capacity, promote relationship-building among communities, and close gaps in services through processes that place value on and supports Indigenous communities’ rights to self-determination and control. The MWP involves collecting and using local data to develop and implement community-specific mental wellness strategies in five First Nations in Ontario.

**Methods:**

The MWP has four key phases. Phase 1 (data collection) includes a community-wide survey to understand MSV issues, service needs and community strengths; in-depth interviews with individuals with lived experiences with MSV issues to understand, health system strengths, service gaps and challenges, as well as individual and community resilience factors; and focus groups with service providers to improve understanding of system weaknesses and strengths in addressing MSV. Phase 2 (review and synthesis) involves analysis of results from these local data sources and knowledge-sharing events to identify a priority area for strategic development based on local strengths and need. Phase 3 (participatory action research approach) involves community members, including persons with lived experience, working with the community and local service providers to develop, implement, and evaluate the MWP to address the selected priority area. Phase 4 (share) is focused on developing and implementing effective knowledge-sharing initiatives. Guidelines and models for building the MWP are shared regionally and provincially through forums, webinars, and social media, as well as cross-community mentoring.

**Discussion:**

MWP uses local community data to address MSV challenges by building on community supports and resilience factors. Drawing on local data and each community's system of formal and informal supports, the program includes sharing exemplary knowledge-to-action models and wellness strategies developed *by* and *for* First Nations people that can be used by other First Nations to identify shared wellness priorities in each community, and determine and execute next steps in addressing areas of main concern.

## Plain English summary

Mental health, substance use, and violence (MSV) are important issues affecting the well-being of Indigenous Peoples in Canada. This paper outlines the protocol for a research-to-action program called the Mental Wellness Program (MWP). The MWP aims to increase community capacity, promote relationship-building between communities, and close gaps in MSV services using methods that value and support Indigenous communities’ rights to self-determination and control. There are four phases to MWP.

1. *Phase 1* includes (a) a community-wide survey to understand MSV issues, service needs and community strengths; (b) interviews with individuals with lived experiences with MSV issues to understand, health system strengths, gaps, and challenges, as well as individual and community sources of resilience; and (c) focus groups with service providers to improve understanding of system weaknesses and strengths in addressing MSV.
2. *Phase 2* involves reviewing results from Phase 1 to identify a priority area for strategic development, based on local strengths and need.
3. *Phase 3* involves community members, including persons with lived experience, working with the community and local service providers to develop, implement, and evaluate the MWP to address the selected priority area.
4. *Phase 4* is focused on developing and implementing effective knowledge-sharing initiatives.

MWP uses local community data to address MSV challenges by building on community supports and resilience factors. Using a model that is developed *by* and *for* First Nations communities will inform wise practices and be of value to other First Nations communities who face similar or related MSV concerns.

## Background

This paper describes the protocol for a research program funded by the Ontario Ministry of Health and Long Term Care’s Health System Research Fund, entitled *Developing a knowledge mobilization model for First Nations mental wellness strategies, building on local knowledge and networks for provincial and national impact* (the Mental Wellness Program). The Mental Wellness Program (MWP) has three important characteristics:

- it builds on strengths and resilience, unlike most previous health research initiatives on or with Indigenous peoples in Canada which have focused on health inequities and deficit-based analyses;
- is consistent with recent influential policies, guidelines, and funding calls related to Indigenous health research in Canada [1–3], it prioritizes Indigenous-led projects, Indigenous self-governance of research data and practices, and capacity building; and
- involves Indigenous communities working together in the spirit of reciprocity, sharing knowledge about challenges and lessons learned.

The overall goal of the MWP is to collect and use local knowledge to establish and address a community-identified priority area related to mental health, substance use, and/or violence (MSV) in five First Nations in Ontario, Canada. The proposal for this project was collaboratively developed by health leaders in two participating First Nations communities, several Centre for Addiction and Mental Health researchers, and both Indigenous and non-Indigenous knowledge users.

### Rooted in colonization: mental health, substance use and violence challenges

The historical, systematic, and systemic colonialism instituted in Canada to assimilate all Indigenous people has caused intergenerational harms that have ongoing and long term effects that are being passed on from one generation to the next [4, 5]. Indigenous people have been severely affected by loss of land, culture and language, grief, chronic trauma, forced assimilation, marginalization, and racist policies [6, 7]. In addition to the effects of historical trauma, Indigenous Peoples experience ongoing stressors, including large socio-economic disparities [8], discrimination, racism, and oppression [5].

Psychological distress related to the effects of colonization have direct links to mental health, substance use and violence issues among Indigenous people [5] that, in turn, contribute to further stress and trauma. Indigenous people are disproportionately affected by mental health challenges compared with non-Indigenous people in Canada [4]. For example, suicide rates among First Nations people is estimated at five to six times higher than non-Indigenous populations [9]. Substance use and addiction are major concerns in many Indigenous communities [10]. One First Nation involved in our study identified prescription drug abuse, illicit drug use, and alcohol use in their top five community challenges while the number one chronic stressor identified by participants was having someone in the family with an alcohol or drug problem [11]. Rates of violence, including partner violence, are also disproportionately high compared with non-Indigenous populations in Canada [12].

Numerous studies have shown that MSV issues are interconnected. For example, a study of Indigenous women who experienced violence found that each additional occasion of heavy drinking by their male partner increased women’s odds of experiencing partner violence by about 17% [12]. Ethnographic interviews suggest that mental health and substance use challenges often result from prior physical, sexual or emotional abuse [13]. Consistent with these findings, in previous research conducted in one First Nation community involved in our study, it was found that harmful drinking and experiences of violence were associated with negative mental health outcomes such as depression, anxiety, and drug use [11].

Given the devastating impact of MSV on individuals, families, and communities, research-based wellness strategies are needed to address these issues, especially co-occurring MSV issues. All too often, funding bodies support short-term and medically-based mental health treatment models that do not consider cultural and historical contexts [14]. While services are often uncoordinated and fragmented, and do not address the need for holistic care for most populations, this need is particularly true for most Indigenous communities in Canada. Meaningful and active involvement from community members is not common in health services planning and consequently, Indigenous people rarely have decision-making power to address MSV challenges in their own communities [15]. Innovative community-driven initiatives with culturally specific Indigenous practices, priorities, and knowledge have proven to show promising results [16]. The MWP model described in the present paper aims to develop community-driven and community-based mental health strategies that are guided by principles of the First Nations Mental Wellness Continuum Framework.

### Rooted in self-determination: the First Nations mental wellness continuum framework

At the core of decolonizing research is a commitment to using research approaches that support Indigenous community self-determination [17]. There are epistemological differences between Indigenous knowledge systems and Western scientific knowledge frameworks and, more often than not, Western-trained researcher expertise is valued more than the expertise of Indigenous leaders, staff, Elders, and knowledge guardians. Smylie [18] proposes that decolonizing research that supports Indigenous self-determination can take the form of Western science being “Indigenized” or where Indigenous science can assert itself in the Western science fields. One framework that supports Indigenous community self-determination in research, policy and program planning is called the *First Nations Mental Wellness Continuum Framework* [19]. This framework was developed in partnership with First Nations people and provides important principles and practical steps for providing comprehensive, culturally relevant, and culturally safe community-based services for First Nations communities. In addition to providing guidance for strengthening programs at federal, provincial and territorial levels, the framework suggests directions for improving mental wellness at the community level. Key elements of the framework that are incorporated into our study include the following.

- *Culture as foundation* recognizes culture as a key social determinant of health and emphasizes approaches that respect, value, and incorporate First Nations’ cultural knowledge, approaches, languages, and ways of knowing into programming and policy development.
- *Community development, ownership and capacity building* supports First Nations communities in building the capacity to shape and develop their own community wellness initiatives, working together in partnerships to address their unique needs and priorities.
- *Quality care system and competent service delivery* supports the development of high-quality and culturally competent services that address the continuum of essential services, including health promotion, prevention, community development and education, early identification and intervention, crisis response, coordination of care and care planning, trauma-informed treatment, support and aftercare.
- *Collaboration with partners* facilitates collaboration and cooperation across sectors and organizations.

The First Nations Mental Wellness Continuum Framework also highlights the importance of:

- building a strong evidence base, founded on First Nations peoples’ knowledge, to inform policy development;
- tracking and communicating progress, with ongoing monitoring, feedback and sharing of knowledge; and
- communities being the primary agents to determine the nature of their mental wellness strategies.

In the context of the MWP, decolonizing research means prioritizing First Nations communities’ needs and voices over researchers and research institutions’ interests in academic productivity and Western science research methods. Throughout all phases of this project, First Nations communities determine the process and desired outcomes of the MWP. For example, a) research activities are governed by a local First Nations Community Advisory Circle; b) culturally relevant and important protocols and practices are followed throughout all phases of the project; c) local and First Nations staff are selected by Community Advisory Circle members and researchers together; d) local research team and Community Advisory Circle members inform and identify the best recruitment, data collection, and knowledge sharing approaches within each community to maximize the level of cultural safety for all community participants; and e) all local research data is owned, controlled, and used in a way that is determined by each participating First Nation community.

The MWP provides a comprehensive understanding of the social contexts of MSV interactions within participating First Nations communities and supports the development of wellness strategies that build on positive factors, such as the strengths and resilience of individuals, families, and communities, to improve access to programs and services that work.

### Building on previous research-to-action initiatives

The MWP is informed by three previous studies. The first study, called *Researching Health in Ontario Communities* (RHOC), used a mobile research laboratory to conduct research in eight communities across Ontario, Canada [20]. The research involved conducting a community survey on MSV issues and the sources of help used for MSV issues. These data were used by researchers and leaders in participating communities to better understand the nature and extent of MSV issues, associations of MSV with stressors and supports and services commonly used by people who have MSV issues [16, 21–23]. The RHOC study also included in-depth interviews with people who had MSV issues and family members of people with MSV issues to examine their experiences accessing and receiving care, including service gaps/barriers and strengths/resilience resources [24].

The second study, called *Five Views on a Journey: Developing a Systems Model of Treatment and Care for Mental Health, Substance Use and Violence Problems* (“Five Views”), built on the RHOC project to better understand how the system of services and informal supports work for people who have MSV issues. Five Views examined the system of services from five perspectives: a) individuals with MSV issues, b) family members of individuals with MSV issues, c) the general population, d) service use data, and e) service providers. The project identified system strengths and challenges to improve systems of care for MSV issues in the local communities and the province, generally. The RHOC and Five Views studies were conducted in eight underserved communities across the province, including two First Nations, Kettle & Stony Point First Nation (“Kettle & Stony Point”) and Aamjiwnaang First Nation (“Aamjiwnaang”).

The third study, titled *Acting Locally to Have a National Impact: A Participatory Action Approach to Addressing First Nations Boys and Men’s Mental Health* (“First Nations Men’s Mental Health Project”), used data from RHOC and Five Views in Kettle & Stony Point, to address the strategic priority of men’s mental health. Findings from RHOC and Five Views indicated that men who had mental health issues had difficulty accessing care and often received inappropriate or ineffective care [25]. The community identified the need for better services addressing boys and men’s mental health. Using participatory action research, a comprehensive healing and wellness program was developed for boys and men within the First Nation community.

Overall, the three studies described above demonstrated the feasibility of collecting rich qualitative and quantitative data to identify and address community-specific priorities. The three studies resulted in significant benefits to participating communities, including: a) the use of local data used to better understand MSV issues for service planning and improvement; b) increased coordination of services through stronger connections among service providers; and c) development of new evidence-based strategies for addressing MSV issues.

The MWP builds on these exemplary initiatives through:

- continued collaboration with Kettle & Stony Point to share knowledge with other First Nations communities regarding the research-to-action process conducted in Kettle & Stony Point;
- continued collaboration with Aamjiwnaang using the data collected there to identify a priority area and build a wellness strategy in that community and participate in knowledge sharing with new communities; and
- new collaborations with three additional First Nations communities that also want to apply this research-to-action approach to develop community-based wellness strategies and share knowledge within and between communities.

## Methods/design

### Aims and objectives

The main aim of this research-to-action program is to develop and implement mental wellness programs (MWP) in First Nations communities using local data and community engagement and to disseminate the findings and strategies regionally and provincially.

The specific objectives are to:

1. use community surveys to better understand how mental health, substance use, and violence interact with each other in each community, including the nature, extent, co-occurrence and links with individual and community strengths and stressors;
2. improve understanding of system weaknesses and strengths in addressing MSV issues by engaging people with lived MSV experiences and local service providers;
3. identify priority areas in each community and develop, implement and evaluate comprehensive, culturally appropriate wellness strategies that address the selected priority areas; and
4. synthesize findings across participating First Nations communities to develop robust recommendations and user-friendly resources for improving the system of services for MSV issues among other First Nations communities at the local, regional, provincial, and national level and implement knowledge translation and exchange activities for adoption/adaptation of the knowledge mobilization model regionally and provincially.

### Overall study design

**Fig. 1:**
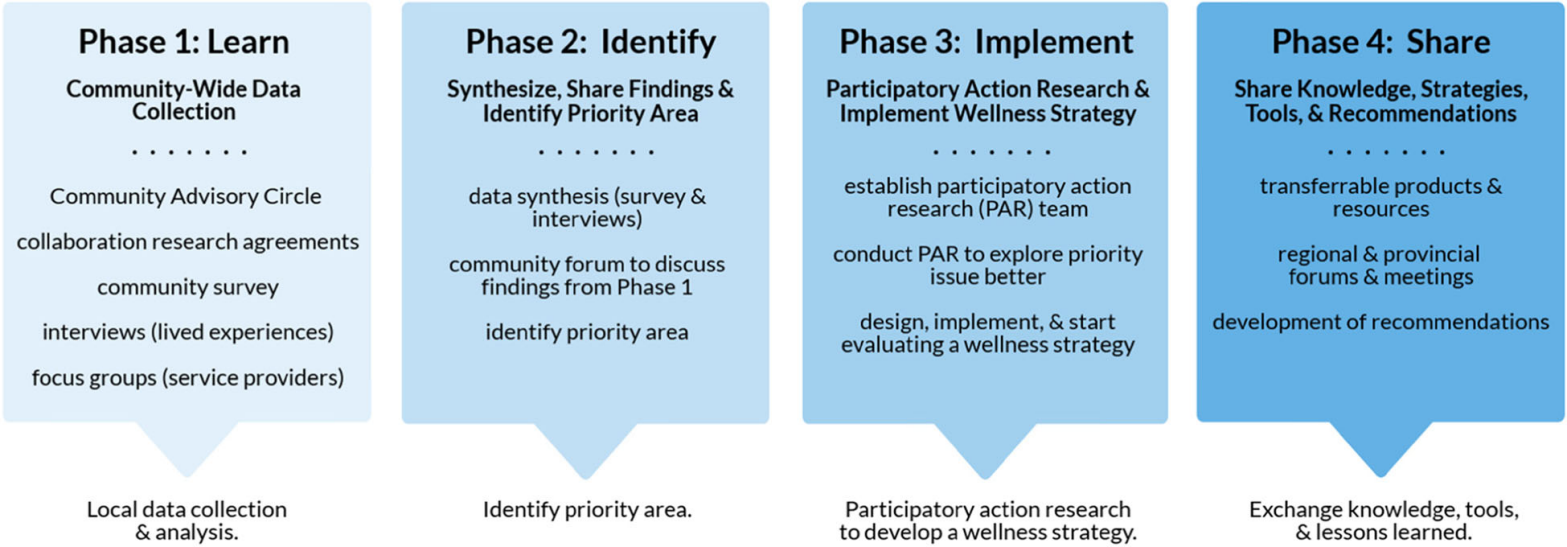
Project Design Overview

Building on the successes and lessons learned in Kettle & Stony Point and Aamjiwnaang, the MWP replicates the process in three other First Nations communities. As shown in Fig. 1 and described in detail below, the MWP involves four main phases: Learn, Identify, Implement, and Share.

### Phase 1 (learn): community-wide data collection

#### Community Advisory Circles

At the outset of the project in each community, a Community Advisory Circle is established to represent the community’s interests, perspectives and concerns. The Community Advisory Circle includes a combination of community leaders, health directors, community health nurses, mental health and addiction support workers, and community members. In each community, the Community Advisory Circle determines the recruitment strategy, reviews and customizes data collection tools, is involved with hiring the Local Research Team, advises on who serves on the Participatory Action Research Team, and works closely with several members of the Centre for Addiction and Mental Health Research Team (Table 1). A Community Health Services Manager or equivalent is allocated release time to Chair the Community Advisory Circle and co-facilitate the research with the Local and Centre for Addiction and Mental Health Research Teams.

**Table 1:** Roles of various teams

Name	Role
Community Advisory Circle (CAC)	Advises and determines how the MWS is conducted within each First Nation community.
Participatory Action Research (PAR) Team	A group of community members who inform and implement the community-specific mental wellness strategy, based on the selected priority area. PAR Teams are largely comprised of community members who have lived experiences in the priority area.
Local Research Team	Hired researchers that work with specific First Nations communities, most often members of the First Nations community where the research is taking place. A Field Coordinator is hired in each community and additional Research Assistants are hired to recruit participants, collect, and help analyze data in Phase 1.
CAMH Research Team	Hired researchers that are involved in the overall MWS project, across all involved First Nations communities.

#### Collaboration research agreements

Collaboration research agreements in each community are developed to articulate: a) the roles and responsibilities of the Community Advisory Circle, Participatory Action Research Team, and Local and Centre for Addiction and Mental Health Research Teams; b) data collection protocols and tools (e.g. survey, interview, and focus groups questions) that are submitted for approval to ethics review board; and c) how research data is managed and governed, following community-identified principles such as the OCAP® [3]. OCAP® stands for the ownership, control, access, and possession of Indigenous research data – ensuring that First Nations communities have a right to determine how data are collected, used, shared, and accessed [3]. Currently, the OCAP® or similar research principles are frequently and rightfully asserted by First Nations communities when planning research projects with outside groups and institutions. The agreements are signed by the designated community leader such as Band Chief or the Health Director and the Vice President of Research at the Centre for Addiction and Mental Health. In the MWP, the Centre for Addiction and Mental Health research staff provide research expertise and administer funding while each participating First Nation’s Community Advisory Circle reviews and makes decisions about data collection tools, project staff, and knowledge sharing activities.

#### Quantitative data collection: community surveys

As indicated above, Phase 1 has already been completed in Kettle & Stony Point and Aamjiwnaang. Three additional First Nations communities in Ontario, Canada, are currently participating in the MWP. The Research Team works with the Community Advisory Circle in each community to develop and implement community surveys that are used to understand MSV issues, service needs, and community strengths. The data collection processes and instruments detailed below are adapted for each community. All data collection procedures are approved by Community Advisory Circle members in each community and the Centre for Addiction and Mental Health Research Ethics Board.

A random sample of 400 community residents aged 18 and older is generated from each community’s band membership list. Advance letters, telephone calls, and door-to-door visits are used to make initial contact with potential participants. A sample population size of 200 to 300 is sufficient for generating estimates of the proportion of a community with a population size of 1000 to 5000 people. The Centre for Addiction and Mental Health mobile laboratory is parked in the community and used to provide office and meeting space for data collection. If desired, participants can complete surveys at other places within the community, so as to respect peoples’ mobility and dignity. Informed consent is obtained prior to beginning the survey and Local Research Team members are available to assist anyone requiring help with completing the survey. Participation is completely voluntary and all data are anonymous and confidential. Participants receive a gift card of $25 as an honorarium for their participation.

As part of the collaborations in Kettle & Stony Point and Aamjiwnaang, a survey was developed including the measures list below and shown in Table 2. In the three other First Nations communities, the survey is reviewed by the Community Advisory Circle to ensure that it meets community-specific needs and interests. Communities may choose to adapt the measures used in previous surveys or add additional measures.

**Table 2:** Inventory of Community Survey Measures

Measures	Brief Description	Source
Demographic information	age, gender, education, employment status, occupation, income, marital status and household composition	
Mental health, substance use/addiction and violence (MSV) issues	depression and anxiety	Composite International Diagnostic Interview (CIDI) Short Form [26]
	hazardous and harmful drinking	Alcohol Use Disorders Identification Test (AUDIT) [27]
	tobacco use, use of licit and illicit drugs	
	aggression victimization and perpetration	The GENACIS (GENder Alcohol and Culture: an International Study) collaboration [28]
	suicide ideation	Canadian Community Health Survey [29]
Stress and trauma	major life events in childhood and adulthood	Measures of stress [30]
	post-traumatic stress disorder	PTSD Checklist [31]
	childhood abuse and neglect	Adverse Childhood Experiences (ACE) [32]
	chronic stress	chronic stressor tool [33]
	racism	Indigenous Racism Experiences (MIRE) scale [34]
	community challenges	First Nations Regional Health Survey [35]
	consequences of colonization and associated negative feelings	Historical Loss Scale and Historical Loss Associated Symptoms Scale [36]
Strengths and supports	social support	Social Provisions Scale (SPS) [37]
	resilience	Connor-Davidson Resilience Scale (CD-RISC) [38]
	social capital	First Nations Social Capital Framework [39]
	social cohesion and control	Social cohesion and social control measures from Project on Human Development in Chicago Neighborhoods [40]
	community strengths	First Nations Regional Health Survey [35]
Service use	services used for MSV	adapted from the Canadian Community Health Survey [41]

##### Analyses of survey data

The analyses include:

- descriptive information about responses e.g., average scores;
- estimates of associations between variables (chi-squared, t-tests, correlations) overall, and for men and women separately;
- linear, logistic and multinomial regression of MSV measures on key explanatory variables (e.g., stress, historical loss and trauma, community strengths and supports) to identify key associations; and
- assessing moderating (e.g., whether associations are different for men and women) and mediating (e.g., extent to which the association of stressors with MSV problems is attenuated by community strengths, supports and resilience) [22, 42].

Community reports are prepared, summarizing the results of all analyses (as done in Kettle & Stony Point and Aamjiwnaang, see [11, 43]) for review and interpretation by members of the Community Advisory Circle, in consultation with the community more broadly as described under Phase 2.

#### Qualitative data collection: interviews and focus groups

##### Interviews with people who have MSV lived experiences and family members

To understand the gaps and challenges as well as strengths and resilience factors for addressing MSV issues in each community, confidential and one-to-one interviews are conducted with 20 people who have MSV challenges as well as 10 family members and caregivers of people with MSV in each community. Participants are asked to describe experiences accessing and receiving help for MSV challenges and recommend ways to improve services. All interviews are audio-recorded and transcribed and reports are prepared for the communities, as done previously [44, 45].

##### Focus groups with service providers

To further understand mental health system gaps and challenges, local service providers from within and outside the community who have been identified by the Community Advisory Circle, the survey and interviews are invited to participate in discussion groups of no more than 15 people. In previous work [46], service providers were eager to be involved and additional discussion groups were held to accommodate all those wishing to participate. Local service providers that are most often working with people who have MSV-related challenges come from a broad range of sectors including health, social services, and corrections among others. Focus groups are conducted or co-led by a member from each of the Community Advisory Circle, the Local Research Team, and the Centre for Addiction and Mental Health Research Team. The discussion focuses on gaps and challenges as well as strengths and resilience factors in each community with exact questions determined by the Community Advisory Circle.

##### Analyses of interview and focus group data

The Community Advisory Circle, Local Research Team, and Centre for Addiction and Mental Health Research Team members work together to identify key emerging themes from the interviews with people of lived experiences and service provider focus groups. Inductive summative analysis [47, 48] is used to organize participants’ experiences and understanding of a) how MSV issues started, b) how and when individuals became aware of their own MSV issues, c) why particular supports were chosen and helpful, and d) the types of supports and services needed to improve the health and wellness of people with MSV in each community.

We use an inductive approach to analyze responses to each focus group question related to a) how the system is working for people who have MSV challenges, b) system weaknesses, challenges and service gaps, c) system strengths that can be built on to enhance programming, and d) how the system can be improved, including how programs and services can be better integrated. An iterative approach to the analysis involves both trained qualitative Centre for Addiction and Mental Health researchers and Local Research Team members in coding and checking for reliability. Once consensus on codes is reached, all transcripts are coded and preliminary findings are reviewed and confirmed by the Community Advisory Circle and Local Research Team before being shared with local First Nations community members. As part of the review and verification process, attention is paid to ways in which the qualitative research findings support and agree with the quantitative survey findings. All findings will be presented to each First Nations community to identify a priority area, described in Phase 2.

### Phase 2 (identify): synthesize, share findings and identify priority area

Community Advisory Circle with the help of the research teams share the findings from the data collection with interested community members in each First Nation at public forum and other sharing events. The aim is to have as much input as possible from the community in identifying the priority area for the project to address.

The Community Advisory Circle may also obtain input and feedback from community members such as Elders and persons with lived experience who may not be able to attend scheduled knowledge sharing events. The Community Advisory Circle may also request further analyses of the data by theResearch Team to help select and define the priority area. By the end of this phase, the Community Advisory Circle and interested community members decide on a mental wellness priority focus.

### Phase 3 (implement): participatory action research and implementation of wellness strategy

Participatory action research is a qualitative research methodology that actively engages the Participatory Action Research Team with other community members in all key aspects of the research and incorporates the knowledge and lived experiences of participants in meaningful ways. The Participatory Action Research Team uses a three-stage process to build the wellness strategy to: a) develop a deeper understanding of the priority area; b) review existing initiatives that might be adopted or adapted to address the priority; and c) implement, monitor and evaluate the MWP.

The Participatory Action Research Team is recruited by the Community Advisory Circle and consists of members of the First Nations with lived experiences related to the priority issue as well as families of people with lived experiences, health care providers, elders and other community members. Members of the Participatory Action Research Team who are not paid by an employer for participating as part of their work, are compensated for their time as a Participatory Action Research Team member. Supported by the Community Advisory Circle and the Centre for Addiction and Mental Health Research Team, the Participatory Action Research Team undertakes a research-to-action process of gaining further knowledge about the priority area and engaging the community and local service providers in building a comprehensive wellness strategy to address the priority area.

The research methodology and methods are determined by the Participatory Action Research Team and may include arts-based methodologies such as Photovoice, as used in Kettle & Stony Point [paper under review]. Photovoice offers a flexible, empowering and collective approach to research [49]. This visual and narrative qualitative research method offers a way to explore life experiences, question deep-seated beliefs, identify power structures, engage in critical reflection, and advocate for community change. Such methods have proven productive in supporting the efforts of First Nations people to challenge community-level effects of historical and ongoing oppression [50]. New and essential information can be acquired using Photovoice, followed by interviews and elder-led sharing circles. Through the use of Photovoice, participants take photos, through sharing their photos during interviews and/or focus groups, contemplate specific issues related to the photos and expand their thinking about those issues issue [paper under review]. Other possible participatory action research methodologies and methods that may be used include interviews, focus groups, story boards, and social media discussions.

The Participatory Action Research Team works together with the Centre for Addiction and Mental Health Research Team to analyze the findings, explore emerging themes, and assess options that address/support the themes. For example, in Kettle & Stony Point, the men who formed the Participatory Action Research Team using Photovoice determined what programs and services were needed for boys and men in the community to improve and maintain overall mental wellbeing [51]. The Participatory Action Research team, together with the Centre for Addiction and Mental Health Research Team and the Community Advisory Circle, then identifies resources needed to accomplish initiatives, short- and long-term outcomes, and develop an evaluation plan. The evaluation plan provides a roadmap for enhancing existing health and social programs, developing new programs, and improving service integration. Each community’s wellness strategy will have their own communication plan that may include media and technology (such as internet, social media, websites, audio/video, local radio and TV, and newspapers) to raise awareness and knowledge regarding the priority issue and the wellness strategy.

### Phase 4 (share): share knowledge, strategies, tools, and recommendations

Knowledge related to the process of identifying, developing, implementing and evaluating the wellness strategies are shared using: a) shareable products/resources that provide other First Nations communities with an overall research-to-action model and step-by-step methods; b) local and regional forums/meetings and a province-wide meeting as well as webinars and other knowledge sharing events; and c) recommendations on how best to implement a regional model for building wellness strategies within First Nations communities.

### Transferrable products and resources

User-friendly and visually engaging products and resources, such as a guidebook and accompanying videos documenting the project’s activities, successes and challenges are being developed and shared. We are developing toolkits and resource manuals that provide hands-on and user-friendly tools to enhance community engagement, build community capacity, mobilize knowledge, collect local data, implement participatory action research, engage local service providers in a process to improve services to their communities, and develop wellness strategies.

Indigenous media outlets such as newspapers, radio stations, television networks, and social media are used throughout the project to share knowledge, products and process from the project. For web-based information, each community decides which community-owned website is used to post, share, and store outputs from this project.

In the MWP’s efforts to promote a spirit of reciprocity between participating communities and community-to-community, targeted efforts are being made build relationships through the sharing of programs and services. For example, in the final year of the “First Nations Men’s Mental Health Project” in Kettle & Stony Point, the men focused on program sustainability through the development of the Mishoomsinaang Mentorship Program, the collaborative under which all of the project’s activities currently fall. The Mishoomsinaang Mentorship Program includes activities such as community sweat lodges, solstice ceremonies, and spring and fall fasting camps. Community members from the MWP that participate in these activities benefit from the relationship-building as well as receive traditional teachings and learn about Anishinabe (First Nations group) roles and responsibilities.

### Regional and provincial forums and meetings

To promote and share the program and its resources, the Community Advisory Circle members select members from their Participatory Action Research Team to serve as Wellness Ambassadors who help determine and coordinate various dissemination activities and play a significant role in presenting the project, its process and findings, locally, regionally and nationally. At the local and regional levels, Wellness Ambassadors, Local Research Team members, and Community Advisory Circle members host community information sessions on the MWP process and share results at local health fairs and other health promotion events.

The model for building wellness strategies as well as guidelines for collecting local data, engaging service providers and using the participatory action research process will be shared with other First Nations communities, service providers, knowledge users, decision makers, and community representatives at provincial and nation-wide meetings and webinars. Annually, participating First Nations will share lessons learned, discuss how the community-specific wellness strategy was implemented in each community and develop a regional model for wellness programming.

### Development of recommendations

In the final year of the project and in consultation with key local, regional, and provincial knowledge users and decision makers, the teams will synthesize all data and document conclusions, suggestions and recommendations generated through knowledge sharing initiatives. Recommendations will include a) successful approaches for building wellness strategies in First Nations communities across the province and b) overarching themes and findings that can be used to improve services for mental health, substance use, and violence issues within First Nations communities.

## Discussion

The MWP is based on Smylie’s [18] premise that Western science research methods, such as epidemiological data analysis, can be decolonized by having Indigenous community members and researchers involved in determining what data are collected and analyzed, articulating the contextual relevance of the findings, and deciding how the findings are shared within and outside of the community. By prioritizing and upholding the importance of strong and honest community-researcher relationships, community leaders and other invested community members can assert ways for local Indigenous knowledge and practices to be incorporated into the MWP.

The MWP is led by Indigenous researchers as well as non-Indigenous researchers who have extensive experience working with Indigenous communities, and most importantly, by community leaders and appointed community members on the Community Advisory Circles. Throughout all facets of the program process, methods, analyses, and interpretation of findings, the Mental Wellness Program aims to facilitate and honour a) Indigenous communities’ rights to self-determination, b) local community knowledge and context, and c) relationships between community and researchers.

All researchers involved in this research program recognize that Indigenous communities and nations have a right to self-determine what research is needed and how research is conducted. As such, while the over-arching progression of the study will be similar in each First Nations community, all phases are discussed and decided by community-appointed members. While some communities use the community survey measures listed in Table 2, other communities may choose to remove some measures and add others. Similarly, each community may identify a different priority area for Phase 3 and participatory action research methodology to address the priority area.

For research to be useful and valued, knowledge and experiences generated by research must be contextually relevant. The overwhelming majority of health research literature is based on Western knowledge paradigms that do not resonate or hold relevance to Indigenous peoples who have survived and thrived for centuries using sacred, undocumented, or unrecognized knowledge. This program holds community-specific knowledge, knowledge systems, and protocols as central to the ways of conducting research and sharing findings.

Limitations of this study include a) working within a three-year project timeframe; b) excluding youth under age 18 as research participants; c) Community Advisory Circle members may not be able to represent the full range of community member perspectives; and d) finding qualified, short-term, and local First Nations contract staff in a timely manner. The MWP must be completed within a three-year funding period and in First Nations community contexts, it is possible that unexpected events or tragedies in a community will affect many community members, causing project delays and interruptions, and revisions to community-specific project activities and timelines.

## Conclusion

The MWP generates local community-wide data to inform wellness strategies that are comprehensive and integrative, building on community supports and resilience factors and involving health and social services both within and outside the communities. The project will develop recommendations for improving services for mental health, substance use and violence issues in First Nations communities at local, regional and provincial levels. The program is expected to culminate in exemplary Indigenous-led knowledge sharing and exchange models as well as wellness strategies developed *by* and *for* First Nations communities addressing high priority issues related to mental wellness.

## Data Availability

The datasets generated and analyzed during the current study are not publicly available. All primary and secondary data are owned and governed by each participating First Nations community. Contact Samantha Wells (co-author) to request information.

## Abbreviations

MSV: Mental health, substance use, and violence
MWP: Mental Wellness Project
OCAP: Ownership, control, access, and possession
RHOC: Researching Health in Ontario Communities
